# Crystal structure and Hirshfeld surface analysis of 5-bromo-1,3,4-thia­diazol-2-amine

**DOI:** 10.1107/S2056989026005335

**Published:** 2026-05-22

**Authors:** Batirbay Torambetov, Miyribek Djiemuratov, Abdusamat Rasulov, Mehmet Akkurt, Gizachew Mulugeta Manahelohe, Khudayar I. Hasanov, Punhan J. Jamalov

**Affiliations:** a National University of Uzbekistan named after Mirzo Ulugbek 4 University St Tashkent 100174 Uzbekistan; b Termez University of Economics and Service, 41B Farovon St., Termiz, 190111, Uzbekistan; c Department of Physics, Faculty of Sciences, Erciyes University, 38039 Kayseri, Türkiye; d Department of Chemistry, University of Gondar, PO Box 196, Gondar, Ethiopia; e Azerbaijan Medical University, Scientific Research Centre (SRC), A. Kasumzade St. 14, AZ 1022, Baku, Azerbaijan; f Department of Chemical Engineering, Baku Engineering University, Khirdalan, Hasan Aliyev str. 120, AZ0101, Absheron, Azerbaijan; Universität Greifswald, Germany

**Keywords:** crystal structure, hydrogen bonds, van der Waals inter­actions, Hirshfeld surface analysis

## Abstract

In the crystal of the title compound, C_2_H_2_BrN_3_S, N—H⋯N hydrogen bonds connect the mol­ecules in the form of hydrogen-bonded double-layered ribbons along the *c*-axis direction including *R*^2^_2_(8) and *R*^2^_1_(3) motifs, which extend to double layers in the *bc*-plane through the same hydrogen bonding albeit at different angles. The actual packing between these layers appears to also be a result of weak vdW inter­actions (Br/S, Br/Br) as well as Br–Br repulsion (steric and/or electrostatic). Looking along *b* or slightly deviating from that direction shows that the latter adopt a zipper pattern in order to not get too close to each other. Hirshfeld surface analysis indicates that N⋯H/H⋯N, Br⋯S/S⋯Br, Br⋯Br and H⋯H contacts account for 69.3% of the total contributions to the Hirshfeld surface.

## Abstract 2

In the crystal of the title compound, N—H⋯N hydrogen bonds connect mol­ecules together, forming ribbons connected along the *c*-axis direction. The actual packing between these ribbons is a result of Br—Br repulsion (steric and/or electrostatic) and they form a zipper-like pattern that obstructs each other’s path.

## 1. Chemical context

Similar to other heterocyclic analogues, 1,3,4-thia­diazo­les are widely used in medicinal, structural and coordination chemistry. In fact, the 1,3,4-thia­diazole moiety acts as a core structural component in an array of drug categories such as anti­cancer, analgesic, anti-inflammatory, anti­microbial, anti­viral, anti-epileptic, anti­neoplastic, and anti­tubercular agents (Jain *et al.*, 2013 ▸; Torambetov *et al.*, 2026 ▸). The strong coordination ability of the nitro­gen atoms is well employed in the construction/engineering of metal complexes towards functional materials (Frija *et al.*, 2016 ▸; Khojabaeva *et al.*, 2025 ▸; Mamedov *et al.*, 2006 ▸). Besides its hydrogen-bond acceptor ability, the sulfur atom of the five-membered thia­diazole ring can also behave as a chalcogen bond donor in inter­molecular inter­actions (Gurbanov *et al.*, 2023 ▸; Mahmudov *et al.*, 2021 ▸). Thus, the presence of a sulfur atom gives it promising characteristics for the development of crystal-engineered materials (Maharramov *et al.*, 2011 ▸) as well as bioactive mol­ecules. The design of the thia­diazol moiety with supra­molecular feature facilitating sites can be used as synthetic strategy to enhance their functional properties (Huseynov *et al.*, 2021 ▸; Naghiyev *et al.*, 2023 ▸; Sadikhova *et al.*, 2024 ▸; Nuralieva *et al.*, 2025 ▸). In this work we have synthesized, isolated and structurally characterized 5-bromo-1,3,4-thia­diazol-2-amine, which exhibits various types of inter­molecular inter­actions, both strong and weak, in its packing.
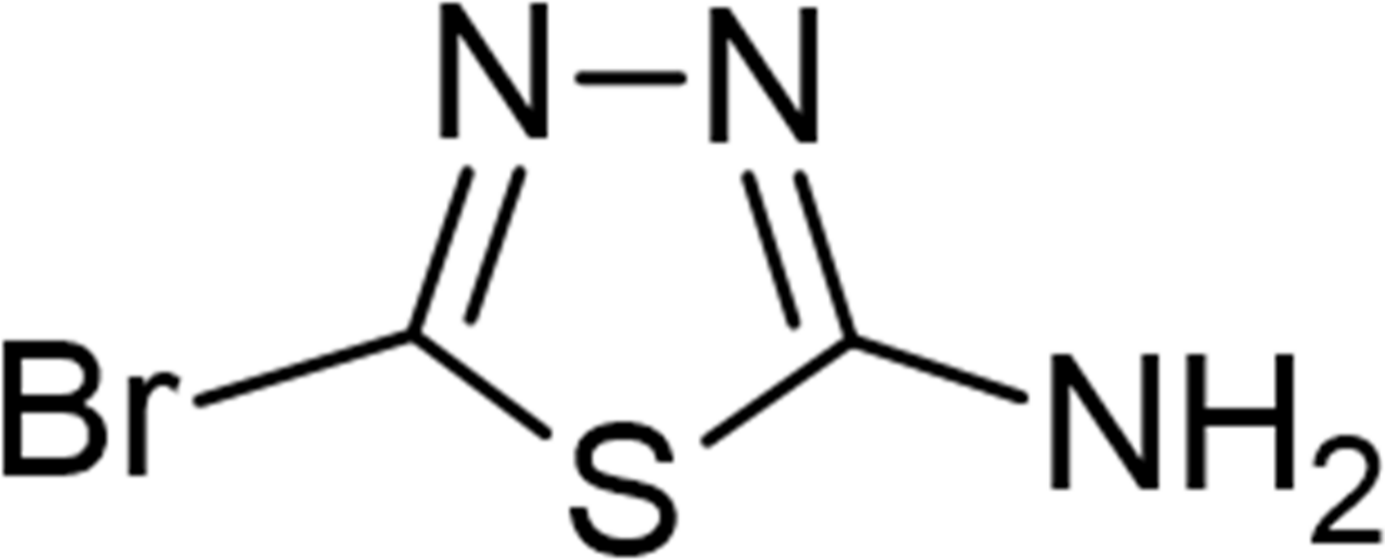


## 2. Structural commentary

**Figure 1:**
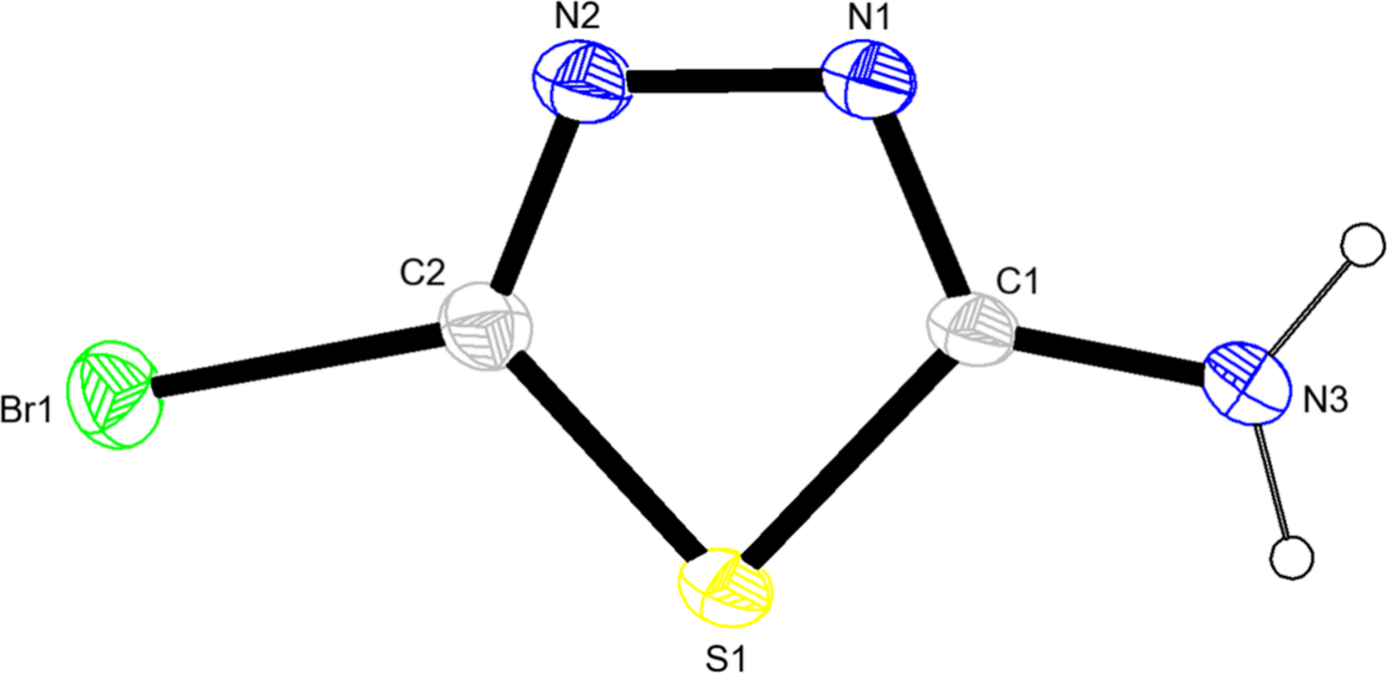
The title mol­ecule with labelling scheme and displacement ellipsoids drawn at the 50% probability level.

The title mol­ecule (Fig. 1 ▸), including the hydrogen atoms, is approximately planar with a r.m.s. deviation of fitted atoms of 0.0220 Å. The maximum deviations from the plane are 0.03 (3) Å for the N3 atom and −0.04 (6) Å and for the H3*A* atom in the opposite direction, indicative of a very subtle pyramidalization of the amine nitro­gen atom N3. The values of the geometric parameters of the mol­ecule are listed in Table 1 ▸ and they appear almost all rather normal. With regard to the C—S—C angle of 86.0 (3)°, this was presumed to be quite reasonable for thia­diazo­les. However, *Mogul* (Bruno *et al.*, 2004 ▸) flagged this as an unusual case and a subsequent search of the Cambridge Structural Database (CSD, Version 6.00, last update April 2025; Groom *et al.*, 2016 ▸) with a focus on this angle was carried out for thia­diazo­les where NH_2_ or a halogen was present on one carbon while the substitution on the other carbon was not specified. This yielded 128 results, of which only four had similar or even more acute angles than the one in the title mol­ecule [ZAJWAM (Makhmudov *et al.*, 2021 ▸) 86.00°; WIXFIS (Tzeng *et al.*, 1999 ▸) 85.17°; WACJIT (Pedregosa *et al.*, 1993 ▸) 85.91°; DEYNII (De Silva *et al.*, 2022 ▸) 85.98°]. The observed acute angle in the title compound is particularly notable because there is no spatial/geometric strain in the title mol­ecule due to its comparably small substituents.

## 3. Supra­molecular features

**Figure 2:**
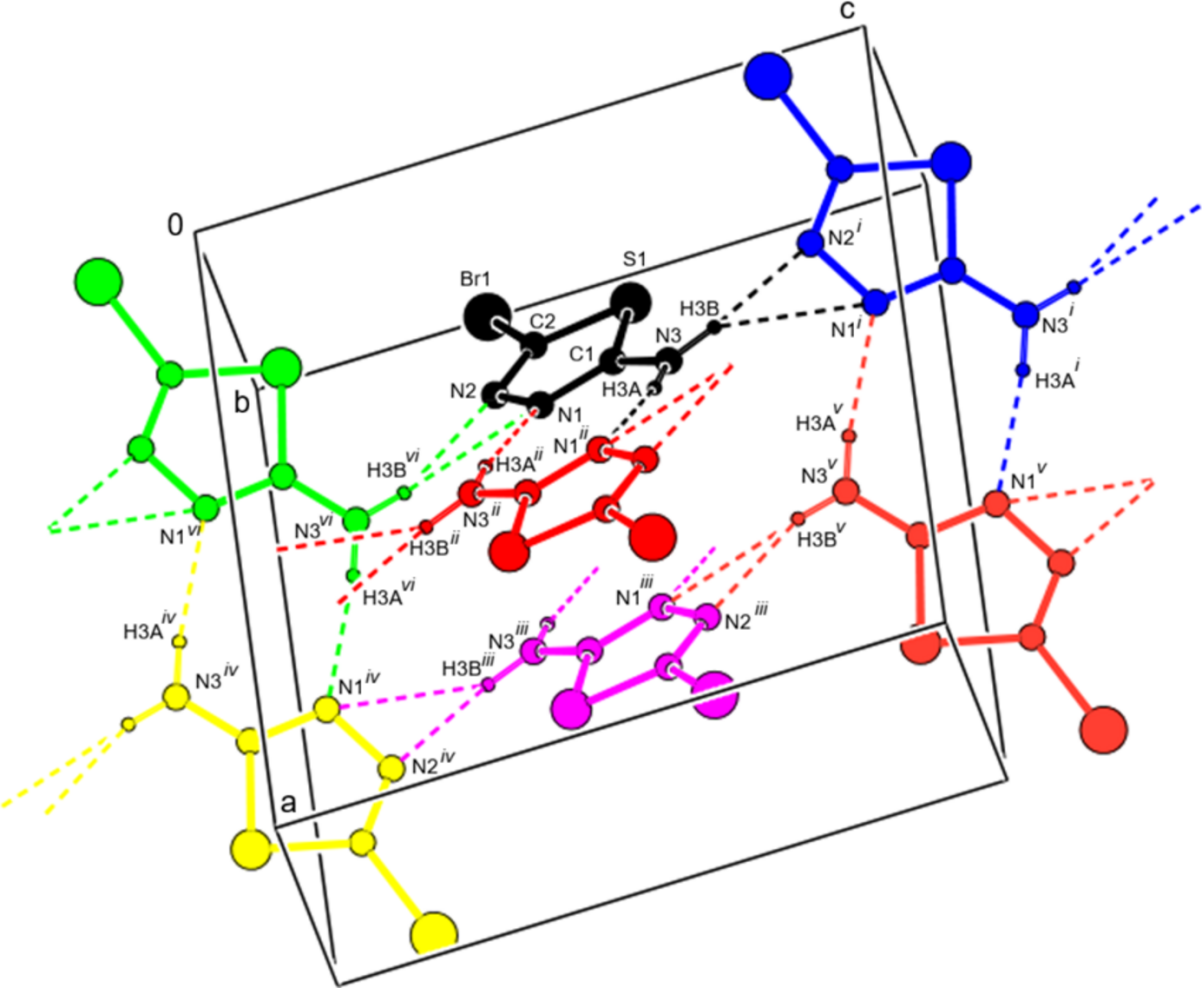
A general view of the inter­molecular N—H⋯N hydrogen bonds in the unit cell. Symmetry codes: (*i) x*, 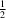 − *y*, 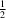 + *z*; (ii) 1 − *x*, − *y*, 1 − *z*; (iii) 1 − *x*, 1 − *y*, 1 − *z*; (iv) 1 − *x*, 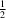 + *y*, 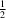 − *z*; (v) 1 − *x*, 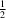 + *y*, 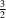 − *z*; (vi) *x*, 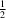 − *y*, − 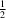 + *z*. Mol­ecules with different equivalent positions are shown in different colours.

**Figure 4:**
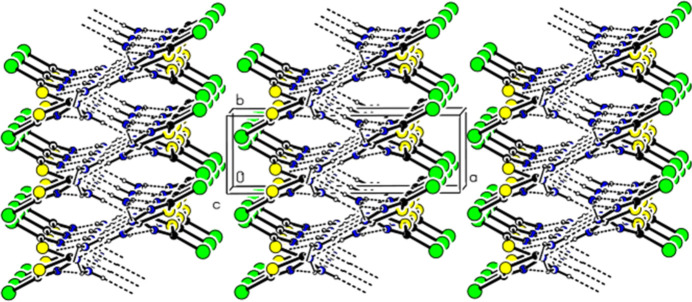
Packing viewed along the *c* axis with inter­molecular hydrogen bonding inter­actions as shown in Fig. 2 ▸.

In the crystal, N—H⋯N hydrogen bonds connect the mol­ecules in the form of hydrogen-bonded ribbons along the *c*-axis direction including 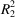
(8) and 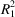
(3) motifs (Table 2 ▸, Figs. 2 ▸ and 3 ▸). The same type of hydrogen bonds (only not in the plane of the mol­ecules) spiral down the *b*-axis direction, forming together with the above described pattern a double layer in the *bc* plane The packing between the hydrogen-bonded layers is further likely also a matter of Br–Br repulsion (steric and/or electrostatic). When viewed along the *b*-direction direction or deviating slightly from this direction, the bromine atoms appear to form a zipper pattern while avoiding becoming too close to each other (Table 3 ▸, Fig. 4 ▸). Their Br⋯Br distances are, in the majority of cases, significantly longer than twice the Br vdW radius [the shortest Br–Br distance is 3.6914 (11) Å for each Br atom’s two neighbours (see below), followed by distances of 4.034 (1) Å or longer]. The shortest distance between Br and S in the crystal is found at 3.8432 (15) Å, which is just about longer than the sum of van der Waals radii. Further, in the crystal packing there are weak or very weak Br⋯Br [3.6914 (11) Å, ΣrvdW(Br⋯Br) = 3.70Å] and S⋯N [3.340 (5) Å, ΣrvdW(S⋯N) = 3.35 Å] inter­actions, but if we consider experimental errors, they cannot be considered as actual halogen or chalcogen bonds, respectively. Therefore, in the crystal, with its packing pattern for inter­actions between the double layers, there is a balance between weak vdW attraction (S/Br and Br/Br) and Br–Br repulsion, which ultimately results in a thermodynamically stable arrangement (Tables 2 ▸ and 3 ▸). C—H⋯π and π–π inter­actions are not observed.

### 3.1. Hirshfeld surface analysis

**Figure 5:**
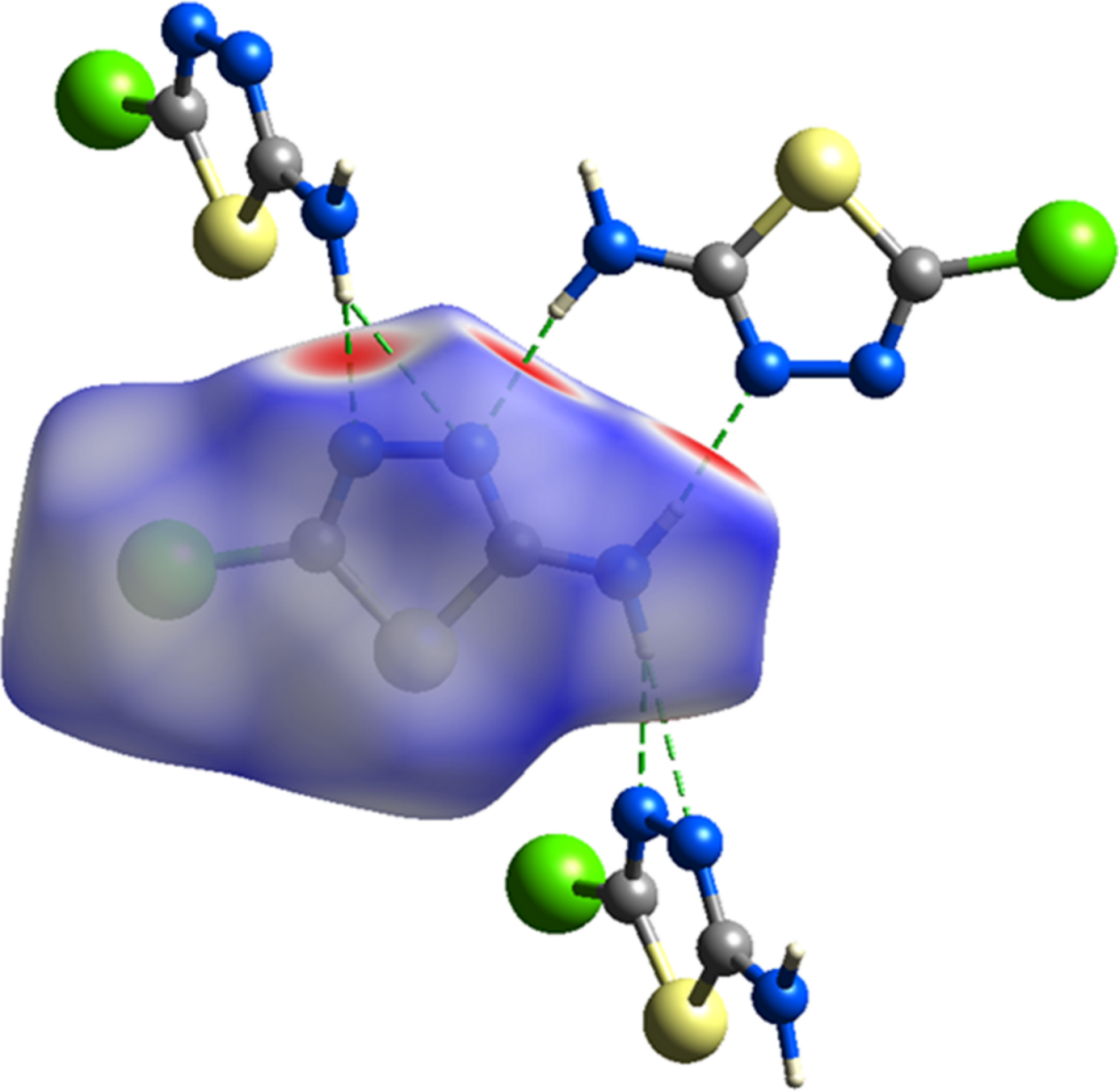
The title compound mapped over *d*_norm_ function on the Hirshfeld surface (colour code. Br: green, C: grey; H: white; N: blue; S: yellow).

A Hirshfeld surface analysis was conducted with *CrystalExplorer* (Spackman *et al.*, 2021 ▸) to observe and qu­antify the inter­molecular inter­actions in the title mol­ecule. The Hirshfeld surfaces were mapped over *d*_norm_ in the range of −0.4897 (red) to +1.0166 (blue) a.u. (Fig. 5 ▸). The red regions are attributed to the N3—H3*A*⋯N1, N3—H3*B*⋯N1 and N3—H3*B*⋯N2 inter­actions (Table 2 ▸). Therefore, there is an equilibrium in the crystal between strong classical hydrogen bonding, weak van der Waals forces of attraction (S/Br and Br/Br) and repulsion (Br–Br), which ultimately leads to an energetic minimum. This is consistent with the Hirshfeld surface shown in Fig. 5 ▸, where the area around bromine is mostly blue.

**Figure 6:**
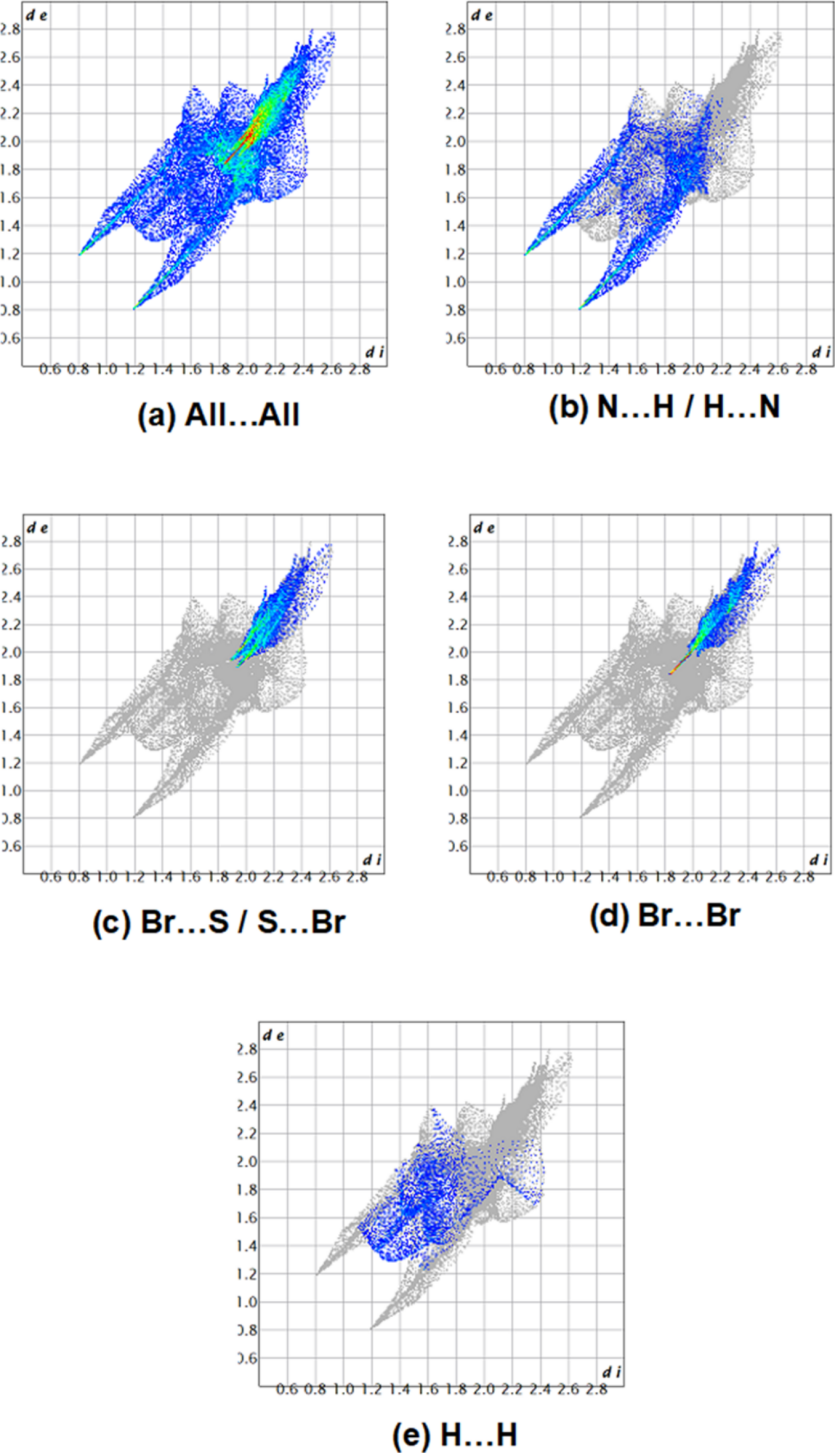
The two-dimensional fingerprint plots, showing (*a*) all inter­actions, and those delineated into (*b*) N⋯H/H⋯N, (*c*) Br⋯S/S⋯Br, (*d*) H⋯H inter­actions; *d*_e_ and *d*_i_ represent the distances from a point on the Hirshfeld surface to the nearest atoms outside (external) and inside (inter­nal) the surface, respectively.

The two-dimensional fingerprint plots demonstrate that the primary contributions to the crystal packing are from N⋯H/H⋯N (25.7%), Br⋯S/S⋯Br (18.1%), Br⋯Br (15.9%) and H⋯H inter­actions (9.6%), as shown in Fig. 6 ▸. Other less notable inter­actions are S⋯N/N⋯S (5.4%), Br⋯C/C⋯Br (4.4%), N⋯N (3.8%), N⋯C/C⋯N (3.0%), S⋯S (2.6%), C⋯C (2.5%), S⋯H/H⋯S (2.9%), S⋯C/C⋯S (2.2%), C⋯H/H⋯C (2.4%) and Br⋯N/N⋯Br (1.6%) inter­actions.

### 3.2. Crystal voids

**Figure 7:**
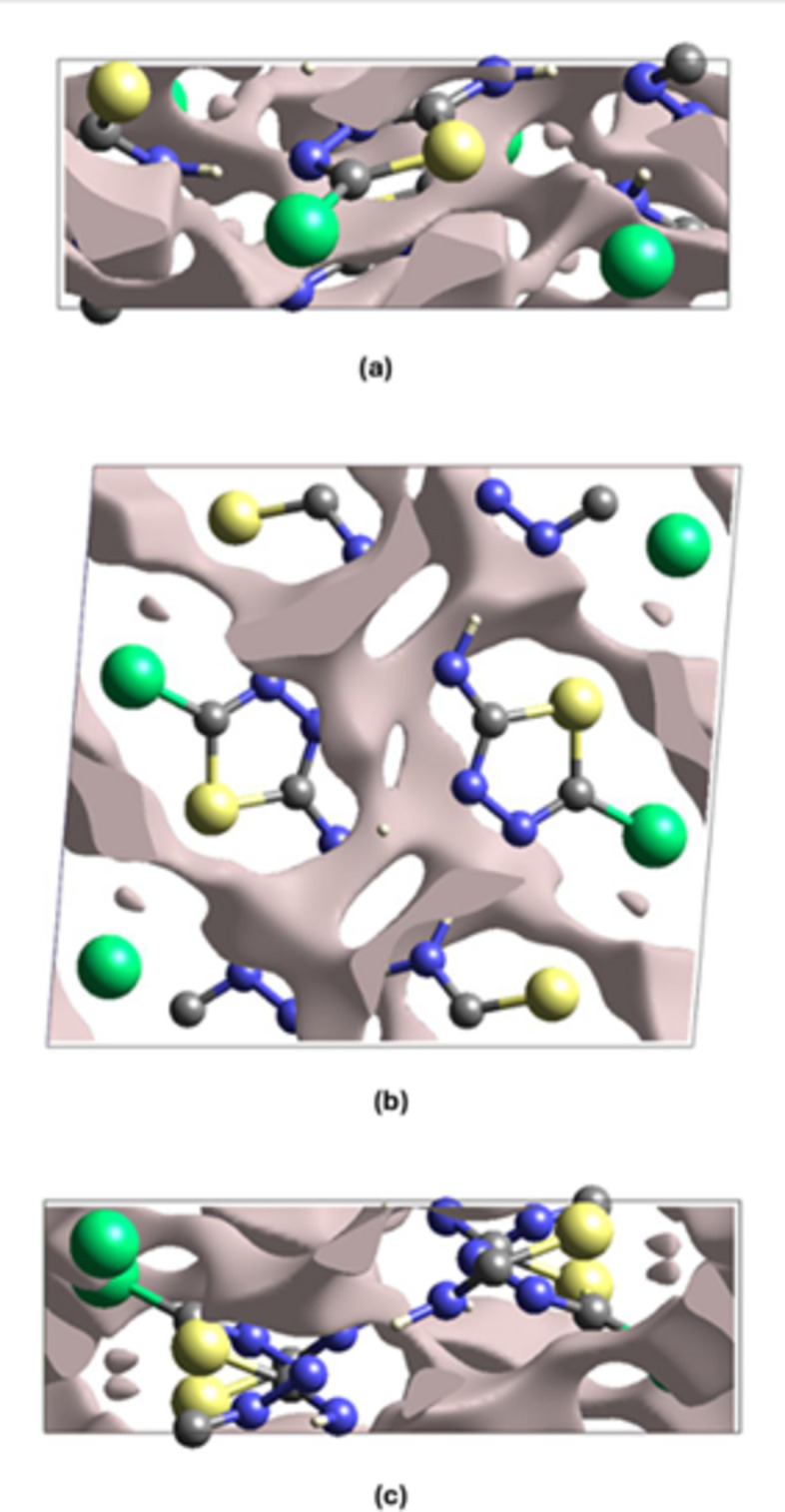
Graphical views of voids in the crystal packing of the title compound along the (*a*) *a*-axis, (*b*) *b*-axis and (*c*) *c*-axis directions.

If the mol­ecules are closely packed and the crystals do not easily break by means of an external mechanical force, then the incorporated void volume is insignificant. The voids in the crystals of the title compound were analysed by summing up the electron densities of all spherically symmetric atoms located within the unit cell (Turner *et al.*, 2011 ▸). The total volume of the crystal voids (Fig. 7 ▸) and the percentage of free space in the unit cell were calculated to be 38.37 Å^3^ and 7.25%, respectively, indicating that the crystal packing is quite compact.

## 4. Database survey

A Cambridge Structural Database (CSD, Version 6.00, last update April 2025; Groom *et al.*, 2016 ▸) search for a thia­diazole with any halogen substituent gave only two hits (DEYMUT and DEYNII; De Silva *et al.*, 2022 ▸) emphasizing the rarity of the combination of a halogen and a thia­diazole group as in the title compound. Both of the above structures exhibit a quite notable network of inter­molecular inter­actions. They contain bifurcated S⋯N as well as *X*1⋯*X*2 [*X*1, *X*2 = halogen (*X*1 = *X*2 = I for DEYMUT and *X*1 = I, *X*2 = Br for DEYNII)] contacts. The latter form zigzag packing patterns due to the typical angle observed in halogen bonding when the σ-hole on a halogen atom (in the two structures: Br or I) inter­acts with a free electron pair of a Lewis base (Metrangolo *et al.*, 2008 ▸), which in these two cases is another halogen atom (I).

Considering that the N-bound H atoms are refined relatively freely resulting in the hydrogen bonds becoming comparably short, another search was carried out for the specific hydrogen-bonding motif between the thia­diazole moieties and adjacent mol­ecules. Excluding metals and aromatic ring structures, when searching for thia­diazo­les exhibiting arrangements similar to the two shorter hydrogen-bonding motifs we observed (*i.e.*, an H⋯N distance range between 2.12 and 2.18 Å), we found only five hits [ESIBOA (Slyvka *et al.*, 2021 ▸), NIYDOO02 (Dani *et al.*, 2013 ▸), VIFRUX01 (Lynch *et al.*, 2001 ▸), XUVPEK (Lynch, 2010 ▸), ZANXUJ (Köysal *et al.*, 2012 ▸)]. The respective observed pattern in the crystal of the title compound is, hence, also notably uncommon.

## 5. Synthesis and crystallization

To a solution of 2-amino-1,3,4-thia­diazole (5 g, 48.45 mmol) in methanol (70 mL), sodium bicarbonate (8.14 g, 96.90 mmol) and bromine (2.5 mL, 48.45 mmol) were added. The reaction mixture was stirred at room temperature until the disappearance of starting material (30–40 minutes). The methanol was removed under vacuum and the crude product was diluted with water (15 mL), filtered, and dried *in vacuo* to give a brown solid, 5-bromo-1,3,4-thia­diazol-2-amine (94%). Colourless crystals suitable for X-ray analysis were obtained by slow evaporation of ethanol solution. Analysis calculated for C_2_H_2_BrN_3_S (*M* = 180.02): C 13.34, H 1.12, N 23.34; found: C 13.30, H 1.10, N 23.31%. ^1^H NMR (300 MHz, DMSO-*d*^6^): *δ* 7.55 (2H). ^13^C NMR (75 MHz, DMSO-*d*^6^) *δ* 170.9 and 124.3.

## 6. Refinement

Crystal data, data collection and structure refinement details are summarized in Table 4 ▸. The N-bound hydrogen atoms were found in difference-Fourier maps and refined relatively freely while constrained with a SADI command at defaults and with *U*_iso_(H) set to 1.2 × *U*_eq_(N).

## Supplementary Material

Crystal structure: contains datablock(s) I. DOI: 10.1107/S2056989026005335/yz2080sup1.cif

Structure factors: contains datablock(s) I. DOI: 10.1107/S2056989026005335/yz2080Isup2.hkl

Supporting information file. DOI: 10.1107/S2056989026005335/yz2080Isup3.cml

CCDC reference: 2555626

Additional supporting information:  crystallographic information; 3D view; checkCIF report

## Figures and Tables

**Figure 3:**
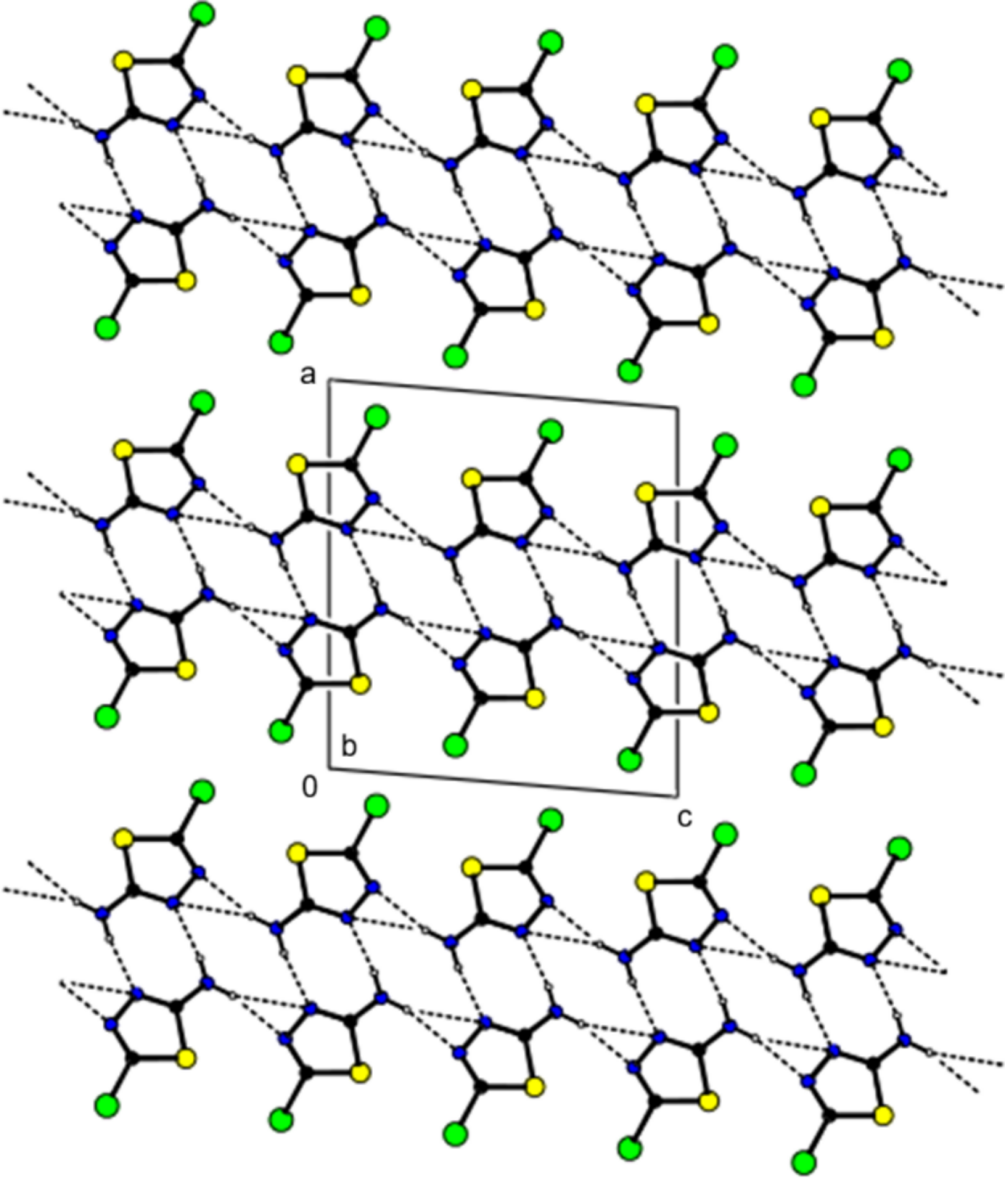
The ribbons connected by hydrogen bonds propagating in the [001] direction viewed along the crystallographic *b* axis. N—H⋯N hydrogen bonds are shown with dashed lines.

**Table 1:** Selected geometric parameters (Å, °)

Br1—C2	1.862 (6)	N1—N2	1.378 (7)
S1—C2	1.732 (6)	N2—C2	1.283 (7)
S1—C1	1.746 (5)	N3—C1	1.339 (8)
N1—C1	1.311 (6)		
			
C2—S1—C1	86.0 (3)	N3—C1—S1	122.3 (4)
C1—N1—N2	112.6 (4)	N2—C2—S1	115.4 (4)
C2—N2—N1	112.5 (5)	N2—C2—Br1	122.7 (4)
N1—C1—N3	124.1 (5)	S1—C2—Br1	121.9 (3)
N1—C1—S1	113.6 (4)		

**Table 2:** Hydrogen-bond geometry (Å, °)

*D*—H⋯*A*	*D*—H	H⋯*A*	*D*⋯*A*	*D*—H⋯*A*
N3—H3*B*⋯N1^i^	0.88 (6)	2.60 (7)	3.398 (7)	150 (8)
N3—H3*A*⋯N1^ii^	0.88 (6)	2.13 (6)	2.997 (7)	171 (7)
N3—H3*B*⋯N2^i^	0.88 (6)	2.14 (6)	2.992 (7)	163 (6)

Symmetry codes: (i) 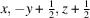; (ii) 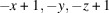.

**Table 3:** Summary of short inter­atomic contacts (Å)

Contact	Distance	Symmetry operation
Br1⋯Br1	3.6914 (11)	−*x*, − 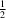 + *y*, 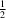 − *z*
S1⋯Br1	3.8432 (15)	−*x*, 1 − *y*, 1 − *z*

**Table 4:** Experimental details

Crystal data	
Chemical formula	C_2_H_2_BrN_3_S
*M*_r_	180.04
Crystal system, space group	Monoclinic, *P*2_1_/*c*
Temperature (K)	293
*a*, *b*, *c* (Å)	12.1005 (13), 4.0336 (5), 10.8832 (14)
β (°)	94.682 (11)
*V* (Å^3^)	529.42 (11)
*Z*	4
Radiation type	Cu *K*α
μ (mm^−1^)	13.20
Crystal size (mm)	0.14 × 0.10 × 0.08

Data collection	
Diffractometer	XtaLAB Synergy, Single source at home/near, HyPix3000
Absorption correction	Multi-scan (*CrysAlis PRO*; Rigaku OD, 2020 ▸)
*T*_min_, *T*_max_	0.256, 0.348
No. of measured, independent and observed [*I* > 2σ(*I*)] reflections	3610, 1012, 915
*R*_int_	0.046
(sin θ/λ)_max_ (Å^−1^)	0.616

Refinement	
*R*[*F*^2^ > 2σ(*F*^2^)], *wR*(*F*^2^), *S*	0.056, 0.175, 1.15
No. of reflections	1012
No. of parameters	70
No. of restraints	1
H-atom treatment	Only H-atom coordinates refined
Δρ_max_, Δρ_min_ (e Å^−3^)	0.95, −0.97

Computer programs: *CrysAlis PRO* (Rigaku OD, 2020 ▸), *SHELXT* (Sheldrick, 2015*a* ▸), *SHELXL* (Sheldrick, 2015*b* ▸), *ORTEP-3 for Windows* (Farrugia, 2012 ▸) and *PLATON* (Spek, 2020 ▸).

## supplementary crystallographic information

### 5-Bromo-1,3,4-thiadiazol-2-amine Crystal data

**Table:**

C_2_H_2_BrN_3_S	*F*(000) = 344
*M**_r_* = 180.04	*D*_x_ = 2.259 Mg m^−^^3^
Monoclinic, *P*2_1_/*c*	Cu *K*α radiation, λ = 1.54184 Å
*a* = 12.1005 (13) Å	Cell parameters from 2101 reflections
*b* = 4.0336 (5) Å	θ = 3.6–70.1°
*c* = 10.8832 (14) Å	µ = 13.20 mm^−^^1^
β = 94.682 (11)°	*T* = 293 K
*V* = 529.42 (11) Å^3^	Block, colourless
*Z* = 4	0.14 × 0.10 × 0.08 mm

### 5-Bromo-1,3,4-thiadiazol-2-amine Data collection

**Table:**

XtaLAB Synergy, Single source at home/near, HyPix3000 diffractometer	1012 independent reflections
Radiation source: micro-focus sealed X-ray tube, PhotonJet (Cu) X-ray Source	915 reflections with *I* > 2σ(*I*)
Mirror monochromator	*R*_int_ = 0.046
Detector resolution: 10.0000 pixels mm^-1^	θ_max_ = 71.9°, θ_min_ = 3.7°
ω scans	*h* = −14→14
Absorption correction: multi-scan (CrysAlisPro; Rigaku OD, 2020)	*k* = −3→4
*T*_min_ = 0.256, *T*_max_ = 0.348	*l* = −13→13
3610 measured reflections	

### 5-Bromo-1,3,4-thiadiazol-2-amine Refinement

**Table:**

Refinement on *F*^2^	1 restraint
Least-squares matrix: full	Hydrogen site location: difference Fourier map
*R*[*F*^2^ > 2σ(*F*^2^)] = 0.056	Only H-atom coordinates refined
*wR*(*F*^2^) = 0.175	*w* = 1/[σ^2^(*F*_o_^2^) + (0.1175*P*)^2^ + 0.3892*P*] where *P* = (*F*_o_^2^ + 2*F*_c_^2^)/3
*S* = 1.15	(Δ/σ)_max_ < 0.001
1012 reflections	Δρ_max_ = 0.95 e Å^−^^3^
70 parameters	Δρ_min_ = −0.97 e Å^−^^3^

### 5-Bromo-1,3,4-thiadiazol-2-amine Special details

**Table:**

Geometry. All esds (except the esd in the dihedral angle between two l.s. planes) are estimated using the full covariance matrix. The cell esds are taken into account individually in the estimation of esds in distances, angles and torsion angles; correlations between esds in cell parameters are only used when they are defined by crystal symmetry. An approximate (isotropic) treatment of cell esds is used for estimating esds involving l.s. planes.

### 5-Bromo-1,3,4-thiadiazol-2-amine Fractional atomic coordinates and isotropic or equivalent isotropic displacement parameters (Å^2^)

**Table:**

	*x*	*y*	*z*	*U*_iso_*/*U*_eq_	
Br1	0.08608 (5)	0.68710 (19)	0.36306 (6)	0.0623 (4)	
S1	0.22468 (11)	0.3687 (4)	0.58975 (12)	0.0534 (5)	
N1	0.3788 (4)	0.2478 (15)	0.4472 (4)	0.0564 (12)	
N2	0.2957 (4)	0.4049 (16)	0.3750 (4)	0.0556 (12)	
N3	0.4221 (4)	0.069 (2)	0.6501 (5)	0.0641 (15)	
H3A	0.484 (5)	−0.02 (2)	0.630 (7)	0.077*	
H3B	0.396 (5)	0.05 (2)	0.723 (6)	0.077*	
C1	0.3547 (5)	0.2121 (15)	0.5617 (5)	0.0476 (12)	
C2	0.2130 (4)	0.4824 (15)	0.4358 (5)	0.0487 (12)	

### 5-Bromo-1,3,4-thiadiazol-2-amine Atomic displacement parameters (Å^2^)

**Table:**

	*U*^11^	*U*^22^	*U*^33^	*U*^12^	*U*^13^	*U*^23^
Br1	0.0439 (5)	0.0734 (7)	0.0685 (6)	0.0037 (2)	−0.0013 (3)	0.0068 (3)
S1	0.0376 (8)	0.0769 (10)	0.0470 (7)	0.0074 (6)	0.0123 (5)	−0.0004 (6)
N1	0.041 (3)	0.085 (3)	0.044 (2)	0.008 (2)	0.0107 (19)	0.000 (2)
N2	0.042 (2)	0.078 (3)	0.048 (2)	0.002 (2)	0.0105 (18)	−0.001 (2)
N3	0.040 (3)	0.104 (5)	0.050 (2)	0.019 (3)	0.0121 (19)	0.009 (3)
C1	0.035 (3)	0.068 (3)	0.041 (2)	0.002 (2)	0.0104 (19)	−0.003 (2)
C2	0.034 (2)	0.065 (4)	0.047 (2)	−0.003 (2)	0.0013 (18)	−0.005 (2)

### 5-Bromo-1,3,4-thiadiazol-2-amine Geometric parameters (Å, º)

**Table:**

Br1—C2	1.862 (6)	N2—C2	1.283 (7)
S1—C2	1.732 (6)	N3—C1	1.339 (8)
S1—C1	1.746 (5)	N3—H3A	0.88 (6)
N1—C1	1.311 (6)	N3—H3B	0.88 (6)
N1—N2	1.378 (7)		
			
C2—S1—C1	86.0 (3)	N1—C1—N3	124.1 (5)
C1—N1—N2	112.6 (4)	N1—C1—S1	113.6 (4)
C2—N2—N1	112.5 (5)	N3—C1—S1	122.3 (4)
C1—N3—H3A	118 (5)	N2—C2—S1	115.4 (4)
C1—N3—H3B	117 (5)	N2—C2—Br1	122.7 (4)
H3A—N3—H3B	124 (7)	S1—C2—Br1	121.9 (3)
			
C1—N1—N2—C2	0.1 (8)	N1—N2—C2—S1	−0.8 (7)
N2—N1—C1—N3	−179.6 (7)	N1—N2—C2—Br1	−178.3 (4)
N2—N1—C1—S1	0.6 (7)	C1—S1—C2—N2	0.9 (5)
C2—S1—C1—N1	−0.9 (5)	C1—S1—C2—Br1	178.5 (4)
C2—S1—C1—N3	179.3 (7)		

### 5-Bromo-1,3,4-thiadiazol-2-amine Hydrogen-bond geometry (Å, º)

**Table:**

*D*—H···*A*	*D*—H	H···*A*	*D*···*A*	*D*—H···*A*
N3—H3*B*···N1^i^	0.88 (6)	2.60 (7)	3.398 (7)	150 (8)
N3—H3*A*···N1^ii^	0.88 (6)	2.13 (6)	2.997 (7)	171 (7)
N3—H3*B*···N2^i^	0.88 (6)	2.14 (6)	2.992 (7)	163 (6)

Symmetry codes: (i) *x*, −*y*+1/2, *z*+1/2; (ii) −*x*+1, −*y*, −*z*+1.
